# Supplementary material for: Venomix: a simple bioinformatic pipeline for identifying and characterizing toxin gene candidates from transcriptomic data
Source: PeerJ. 2018 Jul 31;6:e5361. doi: 10.7717/peerj.5361 (PMC6074769; doi:10.7717/peerj.5361)
Supplement: Supplemental Information 3 [file peerj-06-5361-s003.gz › FinalOutput_E-6/Long-sarafotoxin_1/finaltree.pdf]

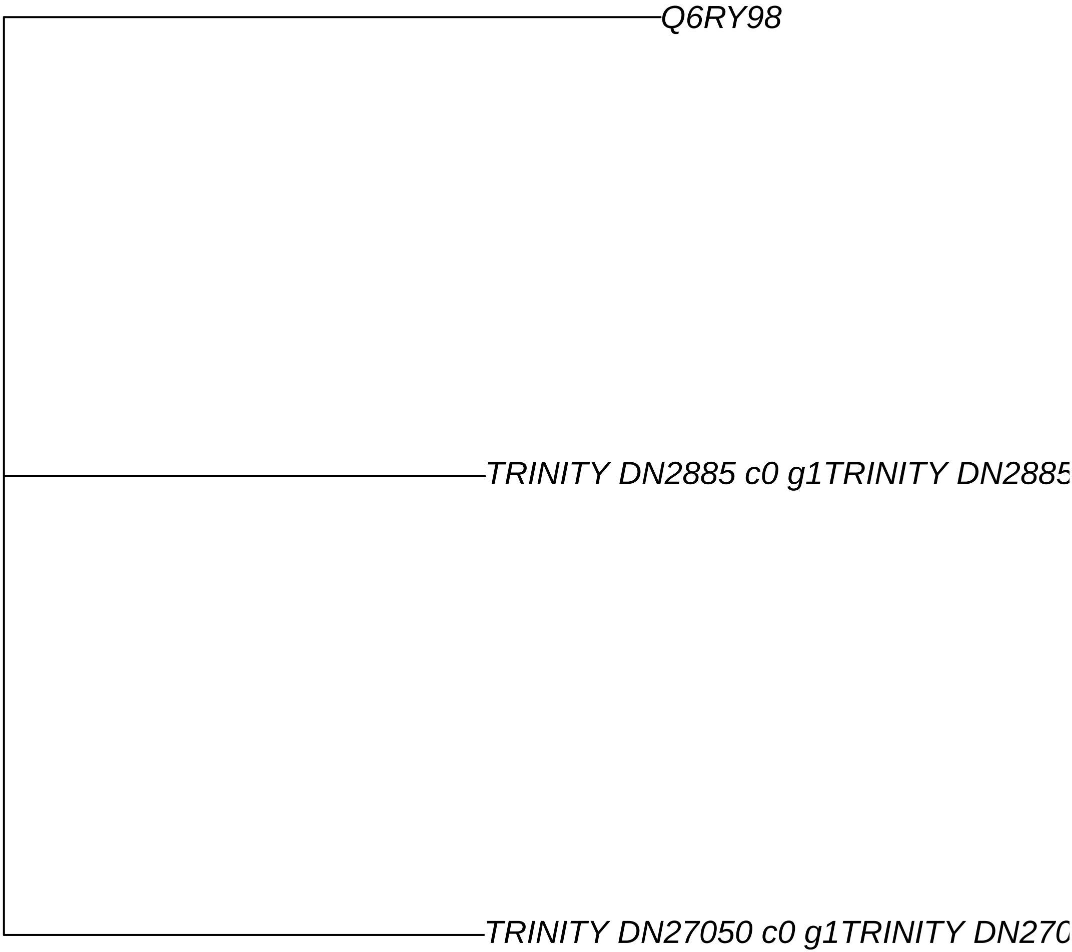
Q6RY98
TRINITY DN2885 c0 g1TRINITY DN2885
TRINITY DN27050 c0 g1TRINITY DN270
